# The burden of disease in Swiss pork production

**DOI:** 10.3389/fvets.2025.1733552

**Published:** 2026-03-05

**Authors:** Giulia Savioli, Dolf Kümmerlen, Beat Thomann

**Affiliations:** 1 Veterinary Public Health Institute, Vetsuisse Faculty, University of Bern, Bern, Switzerland; 2 Swiss Federal Food Safety and Veterinary Office, Bern, Switzerland; 3 Division of Swine Medicine, Department for Farm Animals, Vetsuisse Faculty, University of Zurich, Zurich, Switzerland; 4 Global Burden of Animal Diseases Programme, University of Liverpool, Liverpool, United Kingdom; 5 Institute of Infection, Veterinary and Ecological Sciences, University of Liverpool, Liverpool, United Kingdom

**Keywords:** animal health, burden of disease, economics, GBADs, pork

## Abstract

**Introduction:**

Disease negatively affects the health and productivity of animals, reducing the efficiency and profitability of the livestock sector. Quantifying disease burden in livestock is important to allow appropriate prioritization of diseases and resource allocation in animal health. Although previous studies have quantified costs due to single causes of disease, a consistent approach to estimating total disease costs and comparing them across a wide range of livestock species and production systems was lacking. The development of the Animal Health Loss Envelope (AHLE) metric within the Global Burden of Animals Diseases (GBADs) programme aimed to address these gaps. In this study, we estimate the impacts of improved health status on pig demographics, and estimate the burden due to all causes of disease (the AHLE) for Swiss pork production.

**Methods:**

Using data from the industry, literature and national databases, a demographic model for Swiss pork production was developed, and gross margin analysis was conducted for production scenarios, including current average conditions in Switzerland, and a simulated, disease-free, Ideal scenario.

**Results:**

We estimated that in absence of disease, 41% fewer sows, 3.5% fewer weaned piglets, and 1.5% fewer reared piglets would be required to produce the same number of slaughter pigs compared to current average production. Gross margins were estimated at CHF 469 per breeding sow and CHF 18 per slaughter pig produced under current average production (CHF1: US$1.25). In the absence of disease, gross margins were predicted to increase by CHF 1,856 per sow and CHF 29 per slaughter pig, compared to current average production. Total population AHLE was valued at CHF 461 million, with CHF 187 million attributable to breeding/rearing and CHF 274 million attributable to fattening.

**Discussion:**

These results support that disease significantly impacts production efficiency in Swiss pork, with possible environmental knock-on effects (e.g., through increased feed-demand and farm waste production), and animal welfare impacts. The total population burden of disease estimated here, equal to around half of the total production value of Swiss pork, is significant. Further research should focus on attribution of disease burden to individual causes, framed within this overall AHLE estimate, allowing prioritization of diseases for management and control.

## 1 Introduction

Across all nations, livestock provide valuable resources including nutritious food, traction power, and fiber. Livestock and their health therefore have an important impact on food security, human health and livelihoods globally. By 2031, pork is expected to account for 38% of global meat production growth (1), and is currently the most consumed meat in Europe (2) and Switzerland (3). Pork production is therefore an important sector for food security and the economy.

In the last decades, the global demand for meat and livestock products has increased significantly, driven by population growth and increasing income in many societies (1). The resulting increased number of livestock required to meet this demand, often accompanied by intensification of production systems, has been accompanied by negative effects of livestock production such as environmental degradation, greenhouse gas emissions, emergence of antimicrobial resistance, and potentially, negative impacts on animal welfare (4, 5). Diseases negatively impact livestock health and productivity, thus increasing the resources required to keep these animals, including economic resources, land, water, and energy. In 2018, the Global Burden of Animal Diseases programme (GBADs) was launched with the aim to quantify the burden of animal disease, guide resource allocation and improve the efficiency and sustainability of livestock production systems globally (6, 7).

Most previous studies on the economic impacts of livestock disease have focussed on single diseases (8). When trying to estimate the total burden of disease by summing individual burdens due to single diseases, there is a risk of overestimation (7, 8). This was highlighted, among other studies, within the Global Burden of Disease (GBD) study on human disease burden, where the sum of deaths attributed to single diseases sometimes significantly exceeded the real population mortality (9). Although valuable for single disease burden estimation, this “bottom-up” approach may fail to take into account comorbidities, potentially resulting in overestimation of total burden (8).

Within GBADs, the Animal Health Loss Envelope (AHLE) metric was developed to account for comorbidities (7). The AHLE is a metric for the total cost of all-cause disease burden in livestock, estimated by comparing the economic output of the current production system to a simulated, disease-free ideal scenario. Mortality is set to zero in this ideal scenario, and other performance parameters are set to maximum levels that can be achieved in the current production and management system (7). The AHLE therefore represents an “envelope” into which all causes of burden due to livestock disease must fit (7). Because the ideal scenario is a simulated perfect state, and essentially impossible to achieve in practice, it does not represent a benchmark to be aimed for by producers. Instead, it is the first step in a wider investigation into how disease burden is distributed across different causes.

Previous studies in Europe have provided AHLE estimates for the United Kingdom (10) and Denmark (11), but there is currently no sector-wide estimate for burden of disease in Swiss pork production. Studies in Switzerland have focussed on the potential economic impacts of transboundary diseases, such as African Swine Fever (12), as well as cost-benefit analyses and economic impact analyses on various endemic diseases, including Enzootic Pneumonia in pigs (13), Foot Rot in sheep (14), and Johne's Disease in dairy cattle (15). Switzerland has a high animal health status, being free of notifiable diseases, including African Swine Fever, Classical Swine Fever and Foot and Mouth Disease. In addition, several swine diseases which are endemic in neighboring countries, such as Enzootic Pneumonia, *Actinobacillus pleuropneumoniae* infection and Porcine Reproductive and Respiratory Syndrome (PRRS) have either been consistently controlled or eliminated in Switzerland (16–18). Perhaps as a consequence, burden of disease has not been investigated extensively in Swiss pork production.

In 2023, the total production value of Switzerland's agricultural sector was around CHF 12 billion, with pork production contributing CHF 0.8 billion (8%) of the total value (19). Swiss swine production is characterized by relatively small, highly interconnected farms, in a highly producer-organized system (16). In 2024, there were approximately 4,700 pig farms in Switzerland, including mixed-species farms (20). Swiss pig farms are relatively small compared to other European countries, with 30% of farms having fewer than 50 animals and only 16% having more than 500 animals (20), compared to average farm sizes of 3,500 pigs in Denmark, and 2,500 pigs in the Netherlands (21). Segmented piglet production is a particular feature found in Switzerland, involving cycling of sows between farrowing and gestation farms–farrowing farms buy sows shortly before farrowing, keeping them until the piglets are weaned and gestation farms buy these sows back, inseminate them and maintain them throughout gestation Farms may carry out a single production stage—breeding, rearing, or fattening—(single-stage farms) or carry out multiple production stages (multi-stage farms). Approximately 93% of pork consumed in Switzerland is produced domestically, with the remaining pork being imported, mainly from Germany and Austria, with a negligeable percentage (< 1%) of Swiss pork being exported (22). Switzerland has high animal welfare standards, with around half of pig farms complying with two subsidy scheme programmes requiring provision of outdoor access (RAUS Scheme), and “animal-friendly housing standards” (BTS Scheme), (23).

Therefore, Swiss pork production is relatively heterogenous, consisting of a network of small, interconnected farms, and is characterized by high animal health and welfare standards. Within this context, an overall metric such as AHLE is helpful to estimate the overall burden across the production sector. Burden of disease estimation is important for proper prioritization and resource allocation in animal health. An overall estimate of disease burden should serve as a basis for further work on burden of individual diseases–these should be set in the context of an overall “envelope” or AHLE. In addition, comparison to AHLE estimates from other countries with different farm sizes and animal health and welfare standards may provide useful insights into the possible effects of these factors on disease burden. Such estimates are currently lacking for Switzerland. This study aims to address this gap by:(1) investigating the livestock demographic impacts of improved health status, and (2) estimating the AHLE for Swiss pork production.

## 2 Materials and methods

We created a model to estimate gross margins per animal and AHLE. The model comprises 4 modules: (1) Input Parameters, (2) Demographic Model, (3) Gross Margins Analysis, and (4) AHLE. The model was created in Microsoft Excel (Microsoft Corporation, Redmond, Washington, USA), and @RISK (Palisade Corporation, Ithaca, New York, USA) was used for stochastic modeling to account for variability and uncertainty of input parameters.

### 2.1 Scenarios and production types

**Figure 1:**
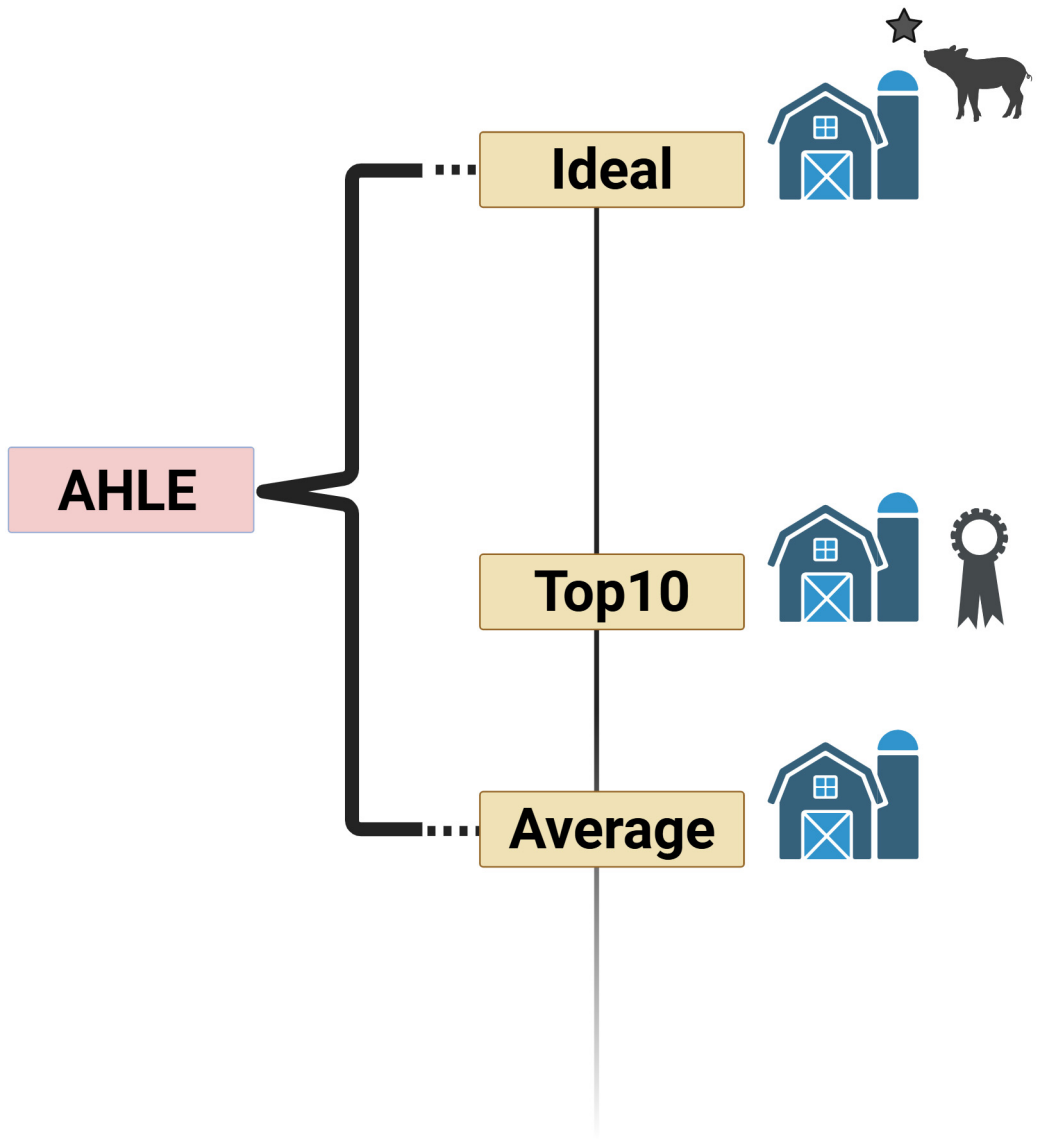
Representation of the three levels of production included in this study. The Animal Health Loss Envelope (AHLE) represents the difference in gross margin between the Ideal and Average scenarios. Created in BioRender. Savioli, G. (2026); https://BioRender.com/0hc0dg3.

Three levels of production performance–or scenarios–were represented: the average Swiss pork production level (Average), the production level of the top 10% producing Swiss pig farms (Top10), and a simulated ideal scenario (Ideal). We calculate the gross margin per animal for all three scenarios, and the gross margin difference between Ideal and Average (the AHLE), at liveweight-, animal-, herd-, and population-level (Figure 1).

For the two empiric scenarios (Average and Top10) production performance was based on piglets weaned per sow per year for breeding farms, and feed conversion ratio (FCR) for fattening herds. The Ideal scenario represents a fictional state of perfect animal health, defined as having zero losses and expenditures due to all causes of disease (including infectious and non-infectious diseases, injuries, and all other disease); within the GBADs framework, animals that are disease-free in this scenario are defined as having “*normality in bodily structure and function that enables the ability to perform physiological functions at normal levels, as long as nutrition and other environmental requirements are provided at adequate levels*” (7).

Based on the available data (see Tables 1, 2, and Supplementary material), and the structure of the Swiss pork industry, we represented the industry as two separate production types: (i) breeding and rearing combined (from now on referred to as breeding/rearing) and (ii) fattening. Distribution of production types between Swiss farms is heterogenous, and detailed farm production-type data is often lacking in national databases (16). Some farms breed and rear piglets for sale to fattening farms, and some farms practice segmented piglet production. Some fattening farms buy reared piglets from farrowing farms, whilst others buy from rearing-only farms. Furthermore, some farms may practice integrated production, carrying out all stages (breeding, farrowing, rearing and fattening) (16). Due to this heterogeneity, to simplify this model we opted for a broad categorization of production types into breeding/rearing and fattening farms.

**Table 1:** Selected input variables used to calculate gross margins and AHLE for breeding/rearing farms.

**Input variable**	**Average^a^**	**Top10^b^**	**Ideal^c^**	**Source**
Gestation length (days)	115	115	115	^a, b, c^Agridea, 2023
Lactation length (days)	31.6	31.6	31.6	^a, b, c^UFA, 2023
Weaning to oestrus interval (days)	22	11	7	^a, b^Calculated, ^c^Assumption based on literature (32).
Piglet weight at weaning (kg)	8	8	8	^a, b, c^Agridea, 2023
Reared piglet weight at sale (kg)	26	26	26	^a, b, c^Assumption, based on UFA, 2023 and Agridea, 2023.
Duration of rearing (days)	42	35	32	^a^Agridea, 2023, ^b^Expert opinion ^c^Calculated
Rearing piglet average daily weight gain (kg)	0.43	0.51	0.57 (0.54–0.59)	^a, b^Calculated ^c^PERT: most likely (min-max)
FCR rearing piglets (kg feed/kg LW)	1.70	1.50	1.35 (1.28–1.43)	^a, b^Expert opinion (Hanspeter Hohl), ^c^PERT: most likely (min-max)
Sow replacement rate (%)	37%	32%	29% (27%−30%)	^a, b^UFA, 2023, ^c^PERT: most likely (min-max)
Litters per sow per year	2.17	2.31	2.34 (2.31–2.37)	^a, b^UFA, 2023, ^c^PERT: most likely (min-max)
Return to oestrus rate (%)	11.1%	3.8%	0%	^a, b^UFA, 2023, ^c^Set to zero.
Piglets weaned per litter	11.7	13.2	17.6	^a, b^UFA, 2023, ^c^Calculated
Piglets weaned per sow per year	25.3	30.6	41.2	^a, b^UFA, 2023, ^c^Calculated
Piglets born alive per litter	13.0	14.3	17.6 (16.8–18.4)	^a, b^UFA, 2023, ^c^PERT: most likely (min-max)
Preweaning mortality (%)	10.5%	6.9%	0%	^a, b^UFA, 2023, ^c^Set to zero.
Rearing mortality (%)	2.0%	1.5%	0%	^a, b^Expert opinion ^c^Set to zero
Farrowing rate (%)	81%	91%	100%	^a, b^UFA, 2023, ^c^Set to maximum.

Sources “Agridea, 2023,” and “UFA, 2023” are production data for the year 2023 supplied on request from the two respective companies Agridea and UFA AG. For formulas used for calculated values, please refer to the Supplementary material. FCR, Feed Conversion Ratio; PERT, PERT Distribution. Superscripts a–c link the data source to the corresponding production scenario (a = Average, b = Top10, c = Ideal).

**Table 2:** Selected input variables used to calculate gross margins and AHLE for fattening farms.

**Input variable**	**Average^a^**	**Top10^b^**	**Ideal^c^**	**Source**
Starting weight (kg LW)	27.1	27.7	27.1	^a, b, c^UFA, 2023
Slaughter weight (kg LW)	115.9	116.8	115.9	^a, b, c^UFA, 2023
Duration of fattening (days)	97	93	83 (79–88)	^a, b^UFA, 2023, ^c^ PERT: most likely (min-max)
Mortality (%)	1.53%	1.46%	0%	^a, b^UFA, 2023, ^c^Set to zero.
FCR (kg feed/kg LW)	2.62	2.35	2.12 (2.00–2.23)	^a, b^UFA, 2023, ^c^ PERT: most likely (min-max)
Average daily weight gain (g/day**)**	917	961	1,064	^a, b, c^Calculated based on starting and slaughter weights, and duration of fattening

Sources “Agridea, 2023,” and “UFA, 2023” are production data for the year 2023 supplied on request from the two respective companies Agridea and UFA AG. LW, Liveweight; FCR, Feed Conversion Ratio; PERT, PERT Distribution. Superscripts a–c link the data source to the corresponding production scenario (a = Average, b = Top10, c = Ideal).

### 2.2 Module 1—Scenario modeling and input parameters

This module comprised production and performance data, pig prices and production costs for pig production in Switzerland. These values were used for the calculations in the Demographic model (Module 2), the gross margin analysis (Module 3), and AHLE calculation (Module 4). Selected input parameter values in the Average, Top10, and Ideal scenarios are provided in Tables 1, 2. An exhaustive table of all input parameters used, including references, can be found in the Supplementary material. Some variables were assumed to remain constant between scenarios, including biologically limited factors such as gestation length and legally dictated factors such as duration of lactation.

We varied veterinary costs for breeding/rearing farms according to the herd size. We assumed that some forms of veterinary care would still be required in the Ideal scenario, namely castration and iron injections for piglets, as these are management measures which would hypothetically still be needed in a state of disease-freedom. Other veterinary costs including herd health visits and vaccination costs are set to zero in the Ideal scenario. The herd health visit, in Average and Top10 is based on a fixed price per farm, plus an additional rate per animal. Therefore, larger farms have a lower veterinary cost per sow.

#### 2.2.1 Average and Top10 scenarios

For the two scenarios based on empiric production data (Average and Top10) we gathered data from a major Swiss livestock feed company (UFA AG), and an independent advisory center for the Swiss agricultural and food industry (AGRIDEA), both for the year 2023. Both these sources provide only average aggregated values, and not individual farm data. Where data was not available, we either estimated values in collaboration with a practicing porcine veterinarian in Switzerland, or calculated values based on available industry and literature data. A detailed explanation of these calculations and sources of all data is available in the Supplementary material.

#### 2.2.2 Ideal scenario

The Ideal scenario is a simulated state of ideal health. Each ideal scenario parameter was defined according to one of six principles (A-F): (A) set to zero (e.g., mortality was assumed to be 0), (B) maximized (e.g., farrowing rate is set to 100%, thus maximized), (C) estimated using Monte Carlo simulation with a PERT distribution (e.g., litters per sow per year), (D) based on the empiric scenario of comparison (e.g., slaughter weight), (E) assumed to remain constant (e.g., gestation length), or (F) calculated based on other inputs. These six methods are described in detail in the Supplementary material.

### 2.3 Module 2—Demographic model

**Figure 2:**
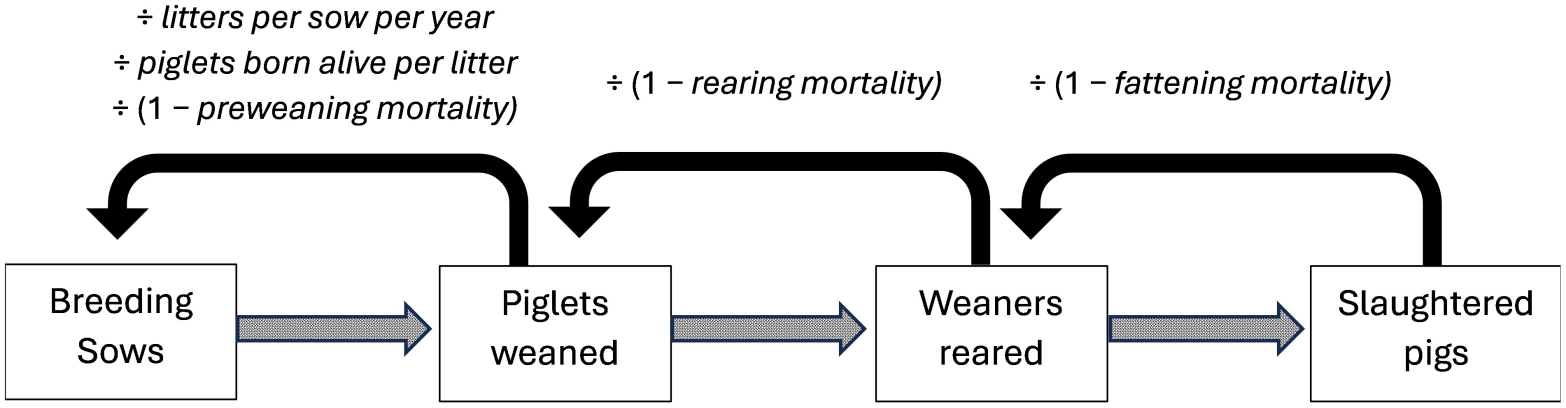
Structure of Module 2: Demographic Model. Solid black arrows represent the back-calculation steps taken to calculate input levels, based on a fixed output, i.e., the number of pigs slaughtered in Switzerland in 2023 (“slaughtered pigs”). Dotted arrows represent the flow of pigs across the production chain.

Within this module, we calculated the number of sows and piglets required to produce a fixed target number of slaughter pigs within 1 year in the Average and Ideal scenarios. The model calculates backwards the number of rearing piglets, weaned piglets and breeding sows required to produce 2,433,981 slaughter pigs [the number of pigs slaughtered in Switzerland in 2023 (20)]. We assume that in the Ideal scenario the number of slaughtered pigs remains the same as in the “real world” scenarios. The multipliers used for the back calculation are: fattening mortality, rearing mortality, litters per sow per year, piglets born alive per litter and pre-weaning mortality (Figure 2). An increase in pork supply is undesirable, as it would lead to reduced demand, thus lowering of pork prices. Therefore, we opt for a fixed number of slaughter pigs across all scenarios and investigate the change in the number of sows and piglets required to achieve this target in the Ideal scenario.

### 2.4 Modules 3, 4—Gross margin analysis and Animal Health Loss Envelope

We calculate gross margins per sow for breeding/rearing farms, and per slaughter pig produced for fattening farms. The gross margin represents the difference between revenues and variable costs for each farm type. For breeding/rearing farms, revenue includes the sale of reared piglets and cull sows. Costs include insemination, total feed, veterinary, and miscellaneous costs. For fattening farms, revenue includes sale of slaughter pigs. Costs include replacement, feed, veterinary and miscellaneous costs.

We then calculate difference in gross margin between the average and ideal scenarios at animal level (AHLE_ANIMAL_) (per breeding sow for breeding/rearing farms, and per slaughter pig produced for fattening farms), and per herd (AHLE_HERD_). The AHLE_HERD_ for breeding/rearing and for fattening farms was based on three different herd sizes: 20, 50, and 150 breeding sows, and 150, 350, and 1,000 slaughter pigs produced, respectively. These sizes are based on expert opinion, and aim to reflect the size of small, medium and large farms commonly found in Switzerland. We also calculate the difference in gross margin per kilogram liveweight (AHLE_KG_) for breeding sows and slaughter pigs, based on liveweight values of 200 kg for sows (24) and 115.9 kg for slaughter pigs (average slaughter weight provide by UFA AG, 2023).

Finally, we estimated the AHLE at population level. First, we calculate the population-level gross margin, by multiplying the number of animals (breeding sows or slaughter pigs produced) by the gross margin per animal calculated in Module 3, for all three scenarios. We used the number of breeding sows (n = 100,878) and slaughtered pigs (n = 2,433,981) in Switzerland in 2023 (20). For breeding/rearing farms, we use the gross margin per breeding sow in 50-sow herd (due to differences in veterinary costs per sow depending on the herd size, the gross margin per sow varies according to the herd size). Then, we calculated AHLE as the difference in population level gross margin between the Average and Ideal scenarios. We calculate separate population-level AHLE (AHLE_POP_) for breeding/rearing farms and for fattening farms, and the combined (total) population AHLE (AHLE_POP_TOTAL_).

### 2.5 Sensitivity analysis

A sensitivity analysis was performed on 13 base input parameters, which include all parameters used to calculate further input parameters, and all parameters set to zero or maximized in the Ideal scenario. The inputs were varied between the Ideal scenario value (maximum used for distribution-based inputs), and the Average scenario value, and simulated over 7 increments for 1,000 iterations.

## 3 Results

### 3.1 Demographic model

**Figure 3:**
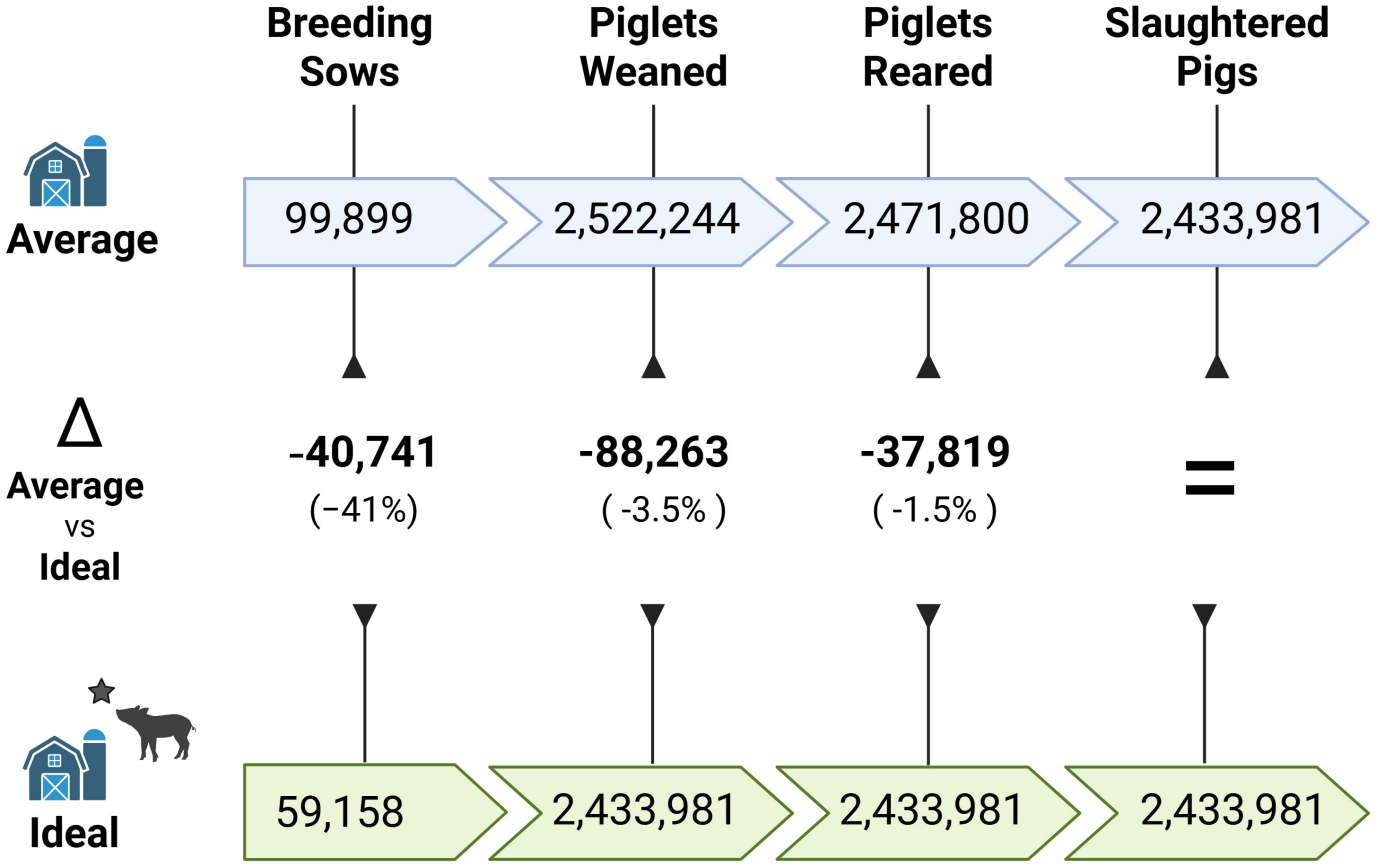
Number of pigs required to produce a target number of slaughter pigs per year, in Switzerland, and the difference in pig numbers between the Average and Ideal scenarios represented as number of pigs (percentage difference compared to Average). The target is based on the number of pigs slaughtered in 2023 (20). For breeding sows in the ideal scenario and Δ breeding sows, mean value outputs of the Monte Carlo simulations are reported. Created in BioRender. Savioli, G. (2026); https://BioRender.com/9gzxjvt.

The demographic model predicted that to produce the same number of slaughter pigs as in 2023, the Ideal scenario would require 41% fewer breeding sows compared to the Average scenario (mean = 40,741 fewer sows; 5^th^-95^th^ percentile range: 38,987–42,448). Additionally, in the Ideal scenario, the number of weaned piglets and reared piglets required would be 3.5% and 1.5% fewer, respectively, compared to the Average scenario (Figure 3). The values for breeding sows are presented as a mean and 5^th^-95^th^ percentile range, because they were calculated based on Monte Carlo-simulated inputs (litters per sow per year, and piglets born alive per litter).

### 3.2 Gross margins and Animal Health Loss Envelope

**Figure 4:**
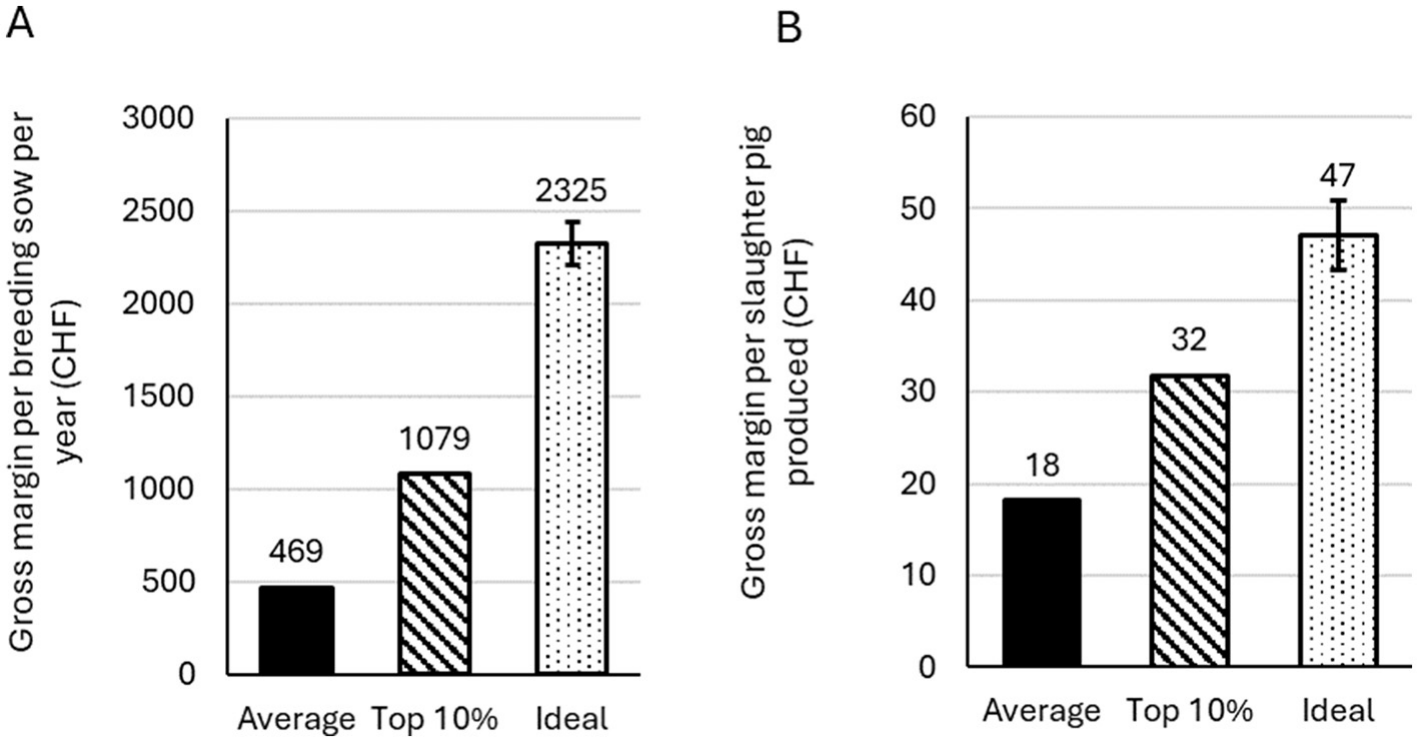
Gross margins per breeding sow per year for breeding/rearing farms **(A)** and per slaughter pig produced for fattening farms **(B)**. Values for breeding herds are based on a herd size of 50 sows. For the Ideal scenario, mean values are presented, with error bars showing the 5^th^ and 95^th^ percentiles of distribution outputs.

**Figure 5:**
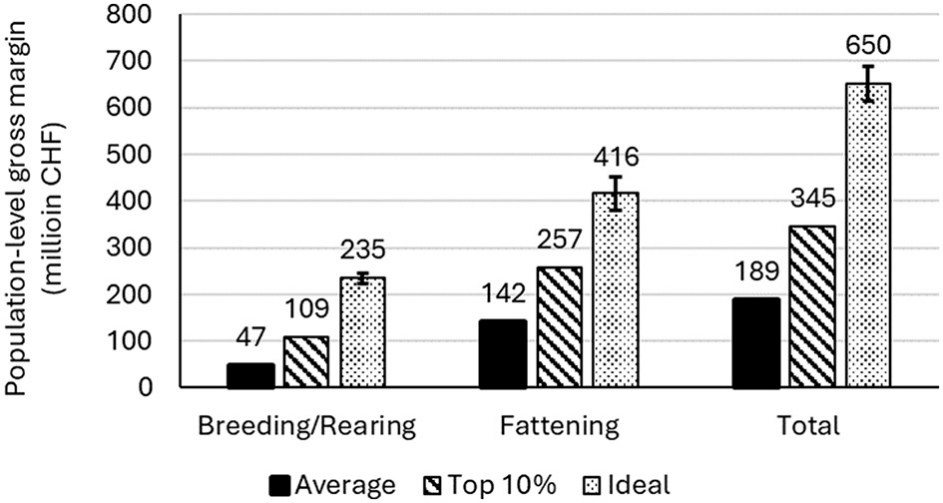
Population level gross margins for Swiss pork production: breeding/rearing farms, fattening farms and all pigs (Total). For the Ideal scenario, mean values are presented, with error bars showing the 5^th^ and 95^th^ percentiles of distribution outputs.

The estimated gross margin per breeding sow per year, for breeding farms with 50 sows in the Average, Top10, and Ideal scenarios was estimated at CHF 469, CHF 1,079, and CHF 2,325 respectively (Table 3, Figure 4A) (exchange rate: CHF 1 = US$ 1.25). For fattening farms, the gross margin per slaughter pig produced in the Average, Top10, and Ideal scenarios was estimated at CHF 18, CHF 32, and CHF 47, respectively (Table 4, Figure 4B). This corresponds to a gross margin difference between Average and Ideal of CHF 1,856 per breeding sow (9.27 CHF/kg LW), and CHF 29 per slaughter pig produced (0.25 CHF/kg LW) (Table 5). We estimated an annual gross margin difference between Average and Ideal of CHF 93,000 for a herd of 50 sows, and CHF 40,000 for a fattening farm producing 350 slaughter pigs per year (Table 5). The total mean population-level AHLE for Swiss pork production was estimated at CHF 461 million, with CHF 187 million and CHF 274 million attributable to breeding pigs and fattening pigs, respectively (Table 5, Figure 5).

**Table 3:** Gross margins for breeding/rearing farms.

**Revenue and variable costs per breeding sow (CHF)**	**Average**	**Top10**	**Ideal**
*Reared Piglets*	2,895	3,544	4,816
*Cull sow*	64	55	50
**Total output**	2,959	3,600	4,866
**Replacement costs**	296	256	230
**Insemination costs**	34	32	29
*Complete feed gestating sow*	505	497	496
*Complete feed lactating sow*	326	347	351
*Starter feed*	39	47	63
*Rearing feed*	591	638	780
*Complete feed replacement gilts*	144	125	112
**Total feed costs**	1,604	1,654	1,803
**Veterinary costs**	117	138	39
*Organizational contributions*	11	11	11
*Ear Tags*	9	11	14
*Cleaning*	13	13	13
*Transport*	201	201	201
*Energy*	50	50	50
*Water*	6	6	6
*Straw Bedding*	144	144	144
*Label inspection costs*	2	2	2
**Total miscellaneous costs**	436	438	441
**Gross margin per breeding sow (CHF), depending on herd size**			
20 breeding sows	463	1,073	2,325 (2,207–2,442)
50 breeding sows	469	1,079	2,325 (2,207–2,442)
150 breeding sows	472	1,082	2,325 (2,207–2,442)
**Gross margin per herd per year (thousand CHF)**			
20 breeding sows	9	21	46 (44–49)
50 breeding sows	23	54	116 (110–122)
150 breeding sows	71	162	349 (331–366)

Ideal scenario values expressed as mean (5^th^-95^th^ percentile). Exchange rate: CHF 1 = US$ 1.25.

**Table 4:** Gross margin for fattening farms.

**Revenue and variable costs per slaughter pig (CHF)**	**Average**	**Top10**	**Ideal**
**Total output**	320	320	320
**Replacement costs**	124	127	122
**Feed costs**	137	124	111
**Veterinary costs**	0.50	0.50	0
*Organizational contributions*	1	1	1
*Cleaning*	6	6	6
*Transport*	16	16	16
*Energy*	3	3	3
*Water*	1	1	1
*Straw bedding*	13	13	13
*Label inspection costs*	1	1	1
**Total miscellaneous costs**	41	41	41
**Gross Margin per slaughter pig produced (CHF)**			
	18	32	47 (43–51)
**Gross Margin per herd per year (thousand CHF)**			
150 slaughter pigs produced	9	16	26 (23–28)
350 slaughter pigs produced	20	37	60 (55–65)
1,000 slaughter pigs produced	58	106	171 (156–186)

Ideal scenario values expressed as mean (5^th^−95^th^ percentile). Exchange rate: CHF 1 = US$ 1.25.

**Table 5:** Animal health loss envelope (AHLE) estimates, per kg liveweight (AHLE_KG_), per animal (AHLE_ANIMAL_), per herd (AHLE_HERD_), and at population level (AHLE_POP_) for breeding/rearing farms and for fattening farms.

**AHLE level**		**Breeding/rearing**		**Fattening**
AHLE_KG_ *(CHF/kg LW)*		9.27 (8.69–9.87)		0.25 (0.22–0.28)
AHLE_ANIMAL_ *(CHF/animal)*		1,856 (1,738–1,973)		29 (25–33)
AHLE_HERD_ *(thousand CHF/herd)*	*Herd size: number of sows*		*Herd size: number of slaughter pigs*	
	*20*	37 (35–40)	*150*	17 (15–19)
	*50*	93 (87–99)	*350*	40 (34–45)
	*150*	278 (260–296)	*1,000*	112 (98–127)
AHLE_POP_ *(million CHF)*		187 (175–199)		274 (238–309)
AHLE_POP_TOTAL_ *(million CHF)*	461 (423–499)			

For breeding/rearing, AHLE_HERD_ is based on the values for a herd with 50 sows. Total population AHLE (AHLE_KG_). Values are expressed as mean (5^th^-95^th^ percentile). AHLE_KG_ and AHLE_ANIMAL_ are for breeding sows and slaughter pigs produced, for breeding/rearing and fattening farms, respectively. LW, liveweight. Exchange rate: CHF 1 = US$ 1.25.

### 3.3 Sensitivity analysis

In total, 13 input parameters (9 for breeding/rearing and 4 for fattening farms) were inspected in the sensitivity analysis for their effect on and the gross margin difference between average and ideal scenarios per animal, for breeding/rearing and fattening farms (AHLE_ANIMAL_), and on the total population AHLE (AHLE_POP_TOTAL_).

**Figure 6:**
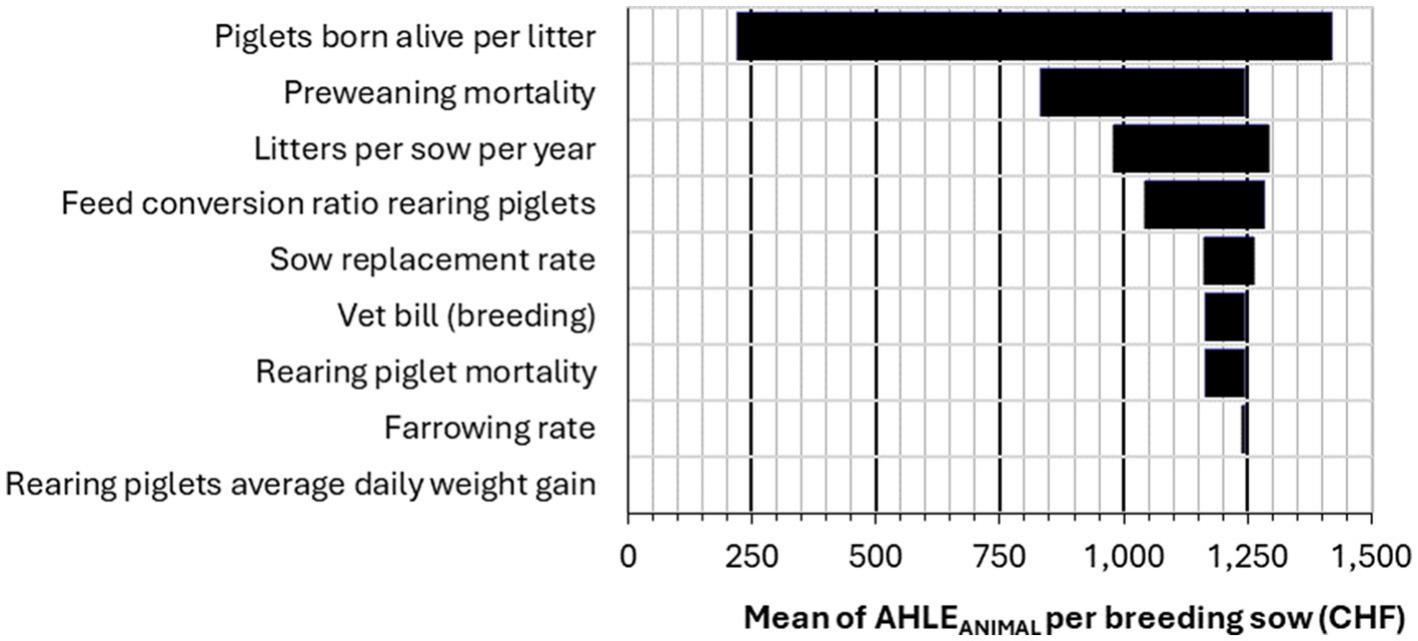
Tornado plot of the sensitivity analysis results for the impact of 9 input parameters on the gross margin difference per breeding sow (Average vs. Ideal scenario), AHLE_ANIMAL_.

**Figure 7:**
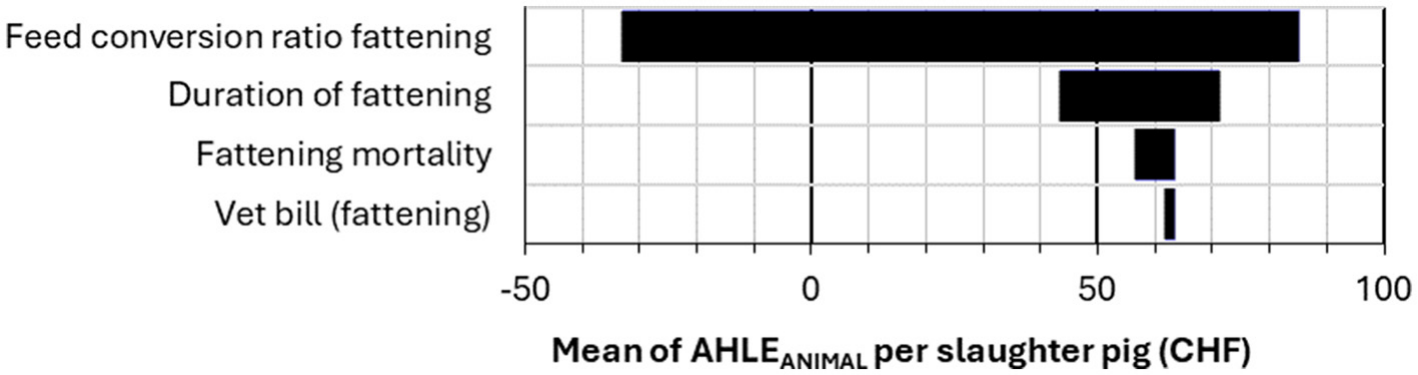
Tornado plot of sensitivity analysis results for the impact of 4 input parameters on the gross margin difference per slaughter pig produced (Average vs. Ideal scenario), AHLE_ANIMAL_.

**Figure 8:**
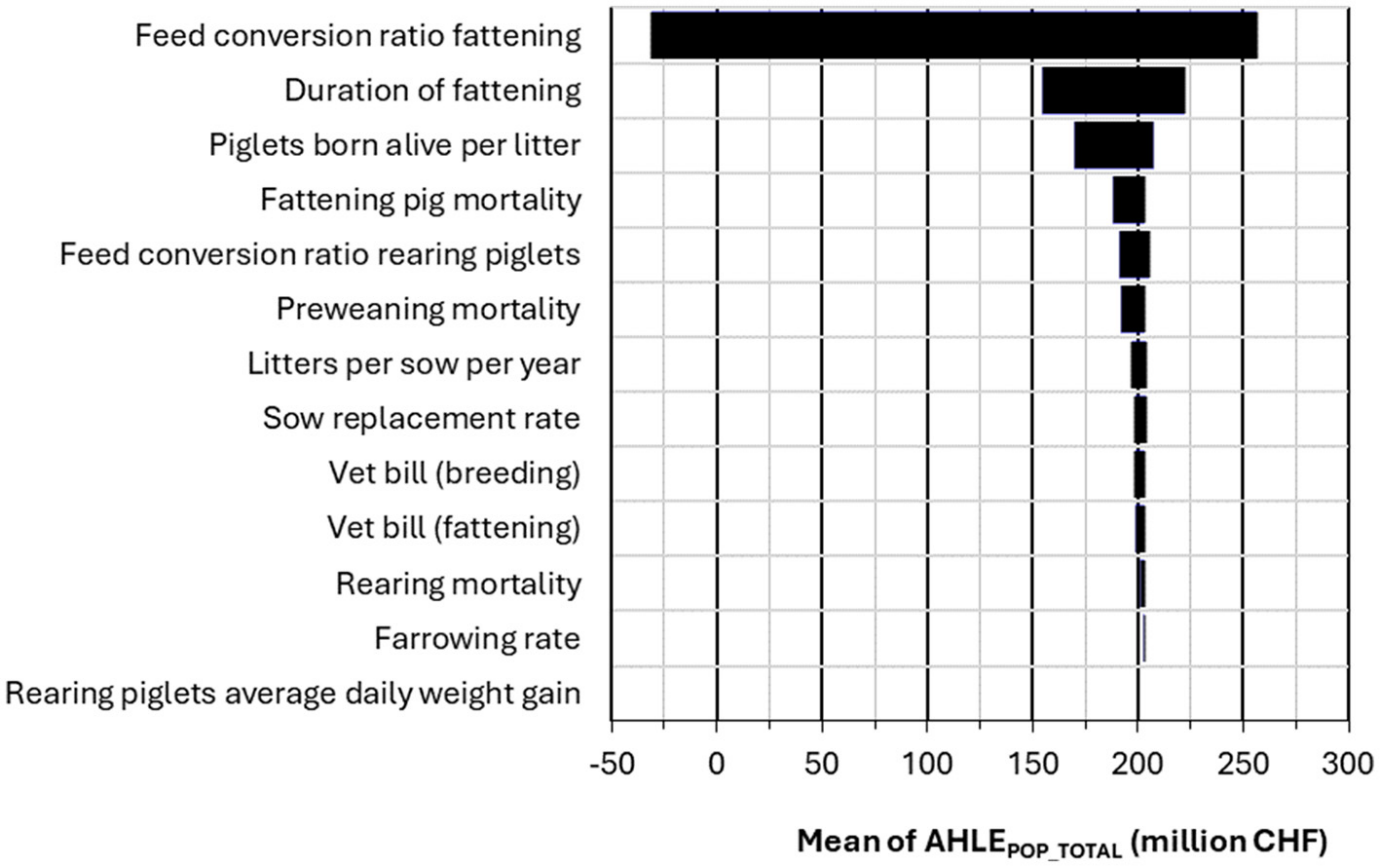
Tornado plot of sensitivity analysis results for the impact of 13 input parameters on AHLE_POP_TOTAL_.

For AHLE_ANIMAL_ per sow, piglets born alive per litter was the most sensitive input parameter (Figure 6). For AHLE_ANIMAL_ per slaughter pig, FCR was the most sensitive input parameter (Figure 7). The sensitivity analysis for the total population AHLE demonstrated that FCR (fattening pig) was the most sensitive parameter by far, followed by duration of fattening and piglets born alive per litter (Figure 8).

## 4 Discussion

We explored the potential effects of improved health status on pig demographics, and assessed the total burden of disease in the pork industry in Switzerland. There is currently a knowledge gap in evaluating burdens of animal diseases and their distribution in value chains (25). Although previous studies have quantified costs due to single causes of disease, a consistent approach to estimating disease costs and comparing them across a wide range of livestock species and production systems was lacking. The development of the AHLE within the GBADs programme aimed to address this gap (7), and in this study we provide estimates for Switzerland.

We estimated that in the absence of disease, pork production would be more efficient than current average production, with fewer animals required overall to produce the same number of slaughter pigs. Since this is one of the first studies carried out within the GBADs framework for swine production, it is not possible to provide extensive comparison with other estimates of AHLE in swine. Our gross margin estimates for of CHF 469 per breeding sow and CHF 18 per slaughter pig produced under current Average production are comparable to industry gross margin estimates for Switzerland (CHF 597 per breeding sow and CHF 18 per slaughter pig, respectively (24)). We also estimated that in the (theoretical) absence of disease, production would be more profitable compared to current average and top performing farms. Our AHLE estimate for slaughter pigs (CHF 0.25, circa 0.31 USD/kg) is comparable to values for overall (population) AHLE estimated for pork production in Denmark in a previous study (0.42 USD/kg) (11).

We estimated a total population AHLE of CHF 461 million, with CHF 187 million attributable to breeding/rearing and CHF 274 million attributable to fattening. This is around half the total population AHLE estimated for the United Kingdom's swine production (GBP 857 million) (10). The UK has twice the number of farms and around three times the number of pigs compared to Switzerland (26), combined with a higher percentage of breeding and rearing farms, which typically showed higher herd-level AHLE (10), partly explaining this difference. For breeding and rearing farms, our combined herd-level AHLE estimate of CHF 278,000 for a herd of 150 sows is higher than the UK estimates of GBP 197,000 (for 250 sows) and GBP 65,000 (for 1,000 rearers), possibly signifying a higher AHLE per animal in the Swiss breeding/rearing system. For fattening farms, our AHLE value was comparable to the UK study, with a herd level AHLE for a 1,000-pig fattening farm of CHF 112,000, similar to the UK value of GBP 127,000.

Switzerland is recognized for having a high animal health status (16, 27). It is free from notifiable swine diseases, including African Swine Fever, Classical Swine Fever, and Aujeszky's Disease (27). In addition, other diseases which are endemic in neighboring countries, including Porcine Reproductive and Respiratory Syndrome (PRRS) and Enzootic Pneumonia have either been eliminated or are consistently under control in Switzerland (16–18). Despite this documented high health status, we estimated a significant burden of disease in Swiss swine production (CHF 461 million), amounting to around half the production value of the swine industry (CHF 900 million) (19). Our burden of disease estimate is comparable to other European countries, despite the absence of certain diseases present in those countries. The cause of this burden in Switzerland may possibly be linked to other endemic diseases and management factors with an impact on herd health. Structural factors and practices such as small farm sizes, and frequent animal movements (for example, in segmented piglet production systems) may be associated with lower biosecurity and increased spread of endemic disease, further contributing to disease burden in Swiss pork. Price levels likely play an important role here, with prices along the entire production chain typically being higher in Switzerland compared to most other countries. Therefore, the same reduction in production efficiency in Switzerland would be associated with higher economic losses compared to other countries. Further investigation, including attribution of disease burden to syndromes or single causes of disease is necessary to elucidate this. The indicators which were found to most strongly impact AHLE in the sensitivity analysis here provide a possible starting point–namely FCR and duration of fattening in fattening pigs, and piglets born alive per litter and weaning mortality in breeding farms. These indicators are likely to be influenced and limited by multiple factors other than infectious diseases, including management practices and farm structure.

This study's findings support that in the absence of disease, the same output (number of slaughter pigs) can be achieved with a lower input. Increased production efficiency could thus result in a reduction in environmental footprint, through lower demand and land-use for feed, reduced environmental pollution from farm waste and reduced greenhouse gas emissions (5, 28). These effects, and their practical implications for livestock production in Switzerland warrant further investigation. Industry-wide improved health status and production efficiency may have a number of further effects, for example on the structure of the pork industry, promoting intensification of pork production, characterized by a reduction in total number of farms coupled with increasing farm size and specialization. This change in the structure is already being observed in Switzerland, with a reduction in the number of farms keeping pigs from 15,347 to 4,726 between the years 2000 and 2024 (29), and the percentage of pig farmers keeping fewer than 200 pigs decreasing from 87% to 65% between the years 2000 and 2019 (22). Nevertheless, farms in Switzerland remain limited in size due to national legislation (30) (maximum 1,500 fattening pigs and 250 breeding sows), setting a boundary to this intensification.

A limitation of our work was the unavailability of private industry data at individual farm-level, with only aggregated data being available. To overcome this, we made assumptions on the production frontiers of the ideal scenario parameters based on literature values or average industry values for Switzerland, thus providing validity for our results in a Swiss-specific context. The model also does not take into account labor costs, or different production labels, both of which might vary with improvements or changes in health status or management system, and would affect farm gross margin–these considerations were deemed beyond the scope of this study. Whilst useful to provide an overall evaluation of the sector-wide disease burden, the AHLE estimates and the Ideal scenario do not provide a benchmark to be aimed for by pork producers. Instead, the Top10 scenario here provides some useful, realistic values to be aimed for by Swiss pork producers. Despite these limitations, our study provides the first AHLE estimate for Swiss pork, accompanied by a demographic model and supported by a sensitivity analysis.

Future research should include attribution studies to investigate which syndromes or individual diseases are responsible for the total disease burden estimated here. Comparison to disease burden attribution studies in countries with similar production systems would be helpful. To investigate the environmental impacts of improved disease status, the demographic model developed here could be expanded upstream to provide insights into how feed demand would change on a population level. Changes in feed consumption are not only linked to animal health, but also to the environmental impact of the industry, including greenhouse gas emissions and land-use (5, 28, 31). Finally, the effect of improved health status on total antimicrobial usage and resistance could also be investigated.

## 5 Conclusions

The aims of this study were to investigate the potential effects of improved health status on pig demographics, and provide an industry-wide estimate of total disease burden for Swiss swine production, both of which were achieved. Although Switzerland has a documented high animal health status, we estimate a significant total burden of disease of CHF 461 million, equivalent to around half the production value of the Swiss swine industry. The results of this study provide insights into the link between disease burden, production efficiency and broader impacts on the economy, sustainable food production, and environmental sustainability. Further research is required to attribute disease burden to individual causes or syndromes, providing guidance for prioritization of animal health interventions and resource allocation.

## Data Availability

The original contributions presented in the study are included in the article/Supplementary material, further inquiries can be directed to the corresponding author.
