# Supplementary material for: The burden of disease in Swiss pork production
Source: Front Vet Sci. 2026 Mar 5;12:1733552. doi: 10.3389/fvets.2025.1733552 (PMC12999446; doi:10.3389/fvets.2025.1733552)
Supplement: Supplementary file 1 [file Data_Sheet_1.pdf]

The Burden of Disease in Swiss Pork Production

Savioli G., Kümmerlen D., Thomann B.

Supplementary Materials

Part 1 – Methods used to Define Ideal Scenario Parameters

Part 2 – Input parameter values and sources

Part 3 – Ideal scenario Monte Carlo simulated parameters.

Part 1 – Methods used to Define Ideal Scenario Parameters

Each ideal scenario parameter was defined according to one of the six principles described below.

**Principle**	**Description**
**A** Set to zero	According to AHLE definitions (1), certain parameters must be set to zero in the ideal scenario. These are mortality, return to oestrus rate, veterinary costs for fattening pigs, veterinary service charge for breeding pigs, and vaccination costs for piglets. For breeding pigs, total veterinary costs are not set to zero, as certain costs are considered inevitably necessary and linked to management rather than health status, namely iron injections and castration of male piglets.
**B** Maximized	Similarly to category A, some parameters are by definition set to the highest possible value given a certain management system. Therefore, we set farrowing rate to 100%. Weaning to oestrus interval was set to 7 days, as according to the available literature (2) this was assumed to be the best achievable value for Swiss management systems.
**C** Monte Carlo Simulation	Seven parameters were estimated using Monte Carlo simulation. The PERT (Programme Evaluation and Review Technique) distribution was used, as it is considered suitable for modelling expert estimates (3). The simulation was run with 10'000 iterations. The parameters simulated in this way were sow replacement rate, litters per sow per year, piglets born alive per litter, average daily weight gain for rearing piglets, FCR for rearing piglets, duration of fattening and FCR for fattening pigs. In all cases except for litters per sow per year, most likely values are set at 10% 'better' than Top10 farms, and minimum and maximum value set at 5% and 15% better than the Top10 scenario (the direction depending on whether a 'better' value is lower or higher for that parameter). For litters per sow per year, the minimum, most likely and maximum values are set at the same value, 1.25% higher and 2.5% better than the Top10 scenario, to remain biologically plausible. Also see Part 3 of these Supplementary Materials.
**D** Based on empiric scenario of comparison	Two parameters, starting weight and slaughter weight for fattening pigs, were set to match the values of the scenario against which the Ideal scenario is being compared. Since we calculated gross margin differences and AHLE, as the difference between Ideal and Average scenarios, the starting weight and slaughter weights for these two scenarios were matched.
**E** Assumed constant	Certain parameters are assumed to remain constant regardless of the scenario, as they are dictated by management factors. Parameters including biologically limited factors such as gestation length and legally dictated factors such as duration of lactation fell into this category. In addition, daily feed consumption per category of swine was assumed to remain constant (however total feed consumption per year varied between scenarios, as animals spent different numbers of days in different categories depending on the scenario). Pig prices, feed prices, and miscellaneous costs such as cleaning, transport and water costs were also assumed to remain the same between different scenarios.
**F** Calculated	Some values were calculated using base input values from the categories above. For example, piglets born alive per sow per year is simply the product of litters per sow per year and piglets born alive per litter.

Part 2 – Input parameter values and sources

Input data values used to calculate gross margins and Animal Health Loss Envelope (AHLE). Sources of the values are marked with [a] for Average scenario, [b] for Top10 scenario and [c] for Ideal scenario. Sources 'Agridea, 2023', and 'UFA, 2023' are production data for the year 2023 supplied on request from the two respective companies Agridea and UFA AG. Abbreviations used: LW: Live weight, DW: Dead weight, dt TS: Decitonnes Dry Matter (Dezitonne Torckensubstanz).

Production parameters	Average[a]	Top10[b]	Ideal[c]	Source
**Fattening Pigs**				
**Starting weight (kg LW)**	27.1	27.7	27.1	[a,b,c]UFA, 2023
**Slaughterweight (kg LW)**	115.9	116.8	115.9	[a,b,c]UFA, 2023
**Dressing percentage (fattening pig)**	79%	79%	79.0%	[a,b,c]Agridea, 2023
**Duration of fattening (days)**	97	93	83<br>(79-88)	[a,b]UFA, 2023,<br>[c] Monte Carlo simulation based on PERT distribution: most likely (maximum-minimum) values indicated.
**Batches per year**	3.2	3.3	3.6	[a]Assumption based on expert opinion,<br>[b,c] Calculated: based on the average scenario, the average number of days between batches of fattening batches was calculated, using the duration of fattening and the batches per year. The result is 17.26 days 'empty', and this value is assumed to remain constant between farms of all production levels, due to management factors. The batches per year in the Top10 and ideal scenarios are calculated as 365/(Duration of fattening + 17.26)
**Mortality (%)**	1.53%	1.46%	0.00%	[a,b]UFA, 2023,<br>[c]set to zero.
**Feed conversion ratio (kg feed/kg LW)**	2.62	2.35	2.12<br>(2.00-2.23)	[a,b]UFA, 2023,<br>[c] Monte Carlo simulation based on PERT distribution: most likely (maximum-minimum) values indicated.
**Hot carcase weight (kg DW)**	91.6	92.3	91.6	[a,b,c]Calculated: (Slaughterweight * Dressing percentage)
**Average daily weight gain (g/day)**	917	961	1064	[a,b,c]Calculated: (Slaughterweight - Starting weight) / Duration of fattening
**Total feed consumption during fattening (kg)**	233	209	188	[a,b,c]Calculated: (Slaughterweight - Starting weight) * Feed conversion ratio
**Breeding pigs**	**Average[a]**	**Top10[b]**	**Ideal[c]**	**Source**
**Breeding sow weight (kg LW)**	200	200	200	[a,b,c]Agridea, 2023

**Dressing percentage (cull sow)**	72%	72%	72%	[a,b,c]Agridea, 2023
**Gestation length (days)**	115	115	115	[a,b,c]Agridea, 2023
**Lactation length (days)**	31.6	31.6	31.6	[a,b,c]UFA, 2023
**Weaning to oestrus interval (days)**	22	11	7	[a,b]Calculated: (365/Litters per sow per year) - gestation length - lacation length, [c]Assumption based on literature (2).
**Batch length (days)**	168	158	154	[a,b,c]Calculated: (Gestation length + Lactation length + Weaning to oestrus interval)
**Piglet weight at weaning (kg)**	8	8	8	[a,b,c]Agridea, 2023
**Reared piglet weight at sale (kg)**	26	26	26	[a,b,c] Assumption, based on UFA, 2023 and Agridea, 2023.
**Duration of rearing (days)**	42	35	32	[a]Agridea, 2023, [b]Expert opinion, [c]Calculated: ((Reared piglet weight at sale - Piglet weight at weaning)/ Rearing piglet average daily weight gain)
**Rearing piglet average daily weight gain (kg)**	0.43	0.51	0.57 (0.54-0.59)	[a,b]Calculated: (Reared piglet weight at sale - Piglet weight at weaning) / Duration of rearing), [c]Monte Carlo simulation based on PERT distribution: most likely (maximum-minimum) values indicated.
**Feed conversion ratio rearing piglets (kg feed/kg LW)**	1.70	1.50	1.35 (1.28-1.43)	[a,b]Expert opinion, [c]Monte Carlo simulation based on PERT distribution: most likely (maximum-minimum) values indicated.
**Daily feed consumption - complete feed gestating sow (kg/sow/day)**	2.9	2.9	2.9	[a,b,c]Agridea, 2023
**Daily feed consumption - complete feed lactating sow (kg/sow/day)**	7.1	7.1	7.1	[a,b,c]Agridea, 2023
**Daily feed consumption - starter feed (kg/piglet/day)**	0.04	0.04	0.04	[a,b,c]Agridea, 2023
**Daily feed consumption - rearing feed (kg/rearer piglet/day)**	0.73	0.77	0.76	[a,b,c]Calculated: (Reared piglet average daily weight gain * Feed conversion ratio rearing piglets)
**Daily feed consumption - complete feed replacement gilts (kg/gilt/day)**	1.6	1.6	1.6	[a,b,c]Agridea, 2023
**Total lactation days per sow per year**	68.6	73.0	73.9	[a,b,c]Calculated: (Lactation length * Litters per sow per year)

**Total days not spent in lactation per sow per year**	296.4	292.0	291.1	[a,b,c]Calculated: (365 - Total lacation days per sow per year)
**Total feed consumption - complete feed gestating sow (kg/sow/365days)**	856	843	841	[a,b,c]Calculated: (Daily feed consumption - complete feed gestating sow * Total days not spent in lactation per sow per year)
**Total feed consumption - complete feed lactating sow (kg/year/365days)**	486	518	524	[a,b,c]Calculated: (Daily feed consumption - complete feed lacatating sow * Total lactation days per sow per year)
**Total feed consumption - starter feed (kg/year/365days)**	30	37	49	[a,b,c]Calculated: (Lactation length * Daily feed consumption - starter feed * Litters per sow per year * Piglets weaned per litter)
**Total feed consumption - rearing feed (kg/year/365days)**	757	818	1000	[a,b,c]Calculated: (Duration of rearing * Daily feed consumption - rearing feed * Piglets reared per sow per year, at 26kg LW)
**Total feed consumption - complete feed replacement gilts (kg/year/365days)**	215	186	167	[a,b,c]Calculated: (Daily feed consumption - complete feed replacement gilts * Sow replacement rate * 365)
**Sow replacement rate (%)**	37%	32%	29% (27%-30%)	[a,b]UFA, 2023, [c]Monte Carlo simulation based on PERT distribution: most likely (maximum-minimum) values indicated.
**Sow service life (years)**	2.7	3.1	3.5	[a,b,c]Calculated: (1 / Sow replacement rate)
**Litters per sow per year**	2.17	2.31	2.34 (2.31-2.37)	[a,b]UFA, 2023, [c]Monte Carlo simulation based on PERT distribution: most likely (maximum-minimum) values indicated.
**Return to oestrus rate (%)**	11.1%	3.8%	0%	[a,b]UFA, 2023, [c]set to zero.
**Piglets weaned per litter**	11.68	13.24	17.6	[a,b]UFA, 2023, [c]Calculated: (Piglets born alive per litter * (1- Preweaning mortality))
**Piglets weaned per sow per year**	25.3	30.6	41.1642	[a,b]UFA, 2023, [c]Calculated: (Litters per sow per year * Piglets weaned per litter)
**Piglets born alive per litter**	13.0	14.3	17.6 (16.8-18.4)	[a,b]UFA, 2023, [c]Monte Carlo simulation based on PERT distribution: most likely (maximum-minimum) values indicated.
**Preweaning mortality (%)**	10.5%	6.9%	0.0%	[a,b]UFA, 2023, [c]set to zero.
**Rearing mortality (%)**	2.0%	1.5%	0.0%	[a,b]Expert opinion, [c]set to zero

**Farrowing rate (%)**	81.0%	91.0%	100.0%	[a,b]UFA, 2023,<br>[c]set to maximum.
**Cull sow hot carcase weight (kg DW)**	144	144	144	[a,b,c]Calculated: (Breeding sow weight * Dressing percentage (cull sow))
**Empty days per cycle**	21.6	11.4	9.5	[a,b,c]Calculated: (365 - Litters per sow per year * (Gestation length + Lactation length))/ Litters per sow per year
**Piglets born alive per year**	28.2	33.0	41.2	[a,b,c]Calculated: (Litters per sow per year * Piglets born alive per litter)
**Piglets reared per sow per year, at 26kg LW**	24.7	30.3	41.2	[a,b,c]Calculated: (Piglets born alive per year * (1 - Preweaning mortality)) * (1 - Rearing mortality)
**Total weight of piglets reared per sow per year (kg LW)**	643	788	1070	[a,b,c]Calculated: (Reared piglet weight at sale * Piglets reared per sow per year, at 26kg LW)
**Culled sow deadweight sold per sow per year (kg DW)**	53.3	46.1	41.5	[a,b,c]Calculated: (Cull sow hot carcase weight (kg DW) / Sow service life)
**Pig prices**	**Average[a]**	**Top10[b]**	**Ideal[c]**	
**Slaughter pig farmgate price (CHF/kg DW)**	3.50	3.50	3.50	[a,b,c]Agridea, 2023
**Reared piglet price at 26kg (CHF/kg LW)**	4.50	4.50	4.50	[a,b,c]Agridea, 2023
**Cull sow price (CHF/kg DW)**	1.20	1.20	1.20	[a,b,c]Agridea, 2023
**Replacement breeding gilt price (CHF)**	800.00	800.00	800.00	[a,b,c]Agridea, 2023
**Production costs**	**Average[a]**	**Top10[b]**	**Ideal[c]**	
Feed and bedding				
**Complete feed fattening pigs (CHF/kg)**	0.59	0.59	0.59	[a,b,c]Agridea, 2023
**Complete feed gestating sow (CHF/kg)**	0.59	0.59	0.59	[a,b,c]Agridea, 2023
**Complete feed lactating sow (CHF/kg)**	0.67	0.67	0.67	[a,b,c]Agridea, 2023
**Starter feed (CHF/kg)**	1.28	1.28	1.28	[a,b,c]Agridea, 2023
**Rearing feed (CHF/kg)**	0.78	0.78	0.78	[a,b,c]Agridea, 2023
**Complete feed replacement gilts (CHF/kg)**	0.67	0.67	0.67	[a,b,c]Agridea, 2023
**Straw consumption - fattening pigs (dt TS)**	0.70	0.70	0.70	[a,b,c]Agridea, 2023
**Straw consumption - breeding pigs (dt TS)**	8.00	8.00	8.00	[a,b,c]Agridea, 2023
**Cost of straw (CHF/dt TS)**	18.00	18.00	18.00	[a,b,c]Agridea, 2023
Veterinary costs - fattening				

**Vet bill per fattening pig per year (CHF)**	0.50	0.50	0.00	[a,b]Expert opinion,<br>[c]set to zero.
Veterinary costs - breeding				
**Veterinary routine visit cost per sow per year - herd size 20 sows (CHF)**	25.00	25.00	0.00	[a,b]Calculated: the flat-rate fee is 200.- per herd, plus 15.- per animal,<br>[c]set to zero.
**Veterinary routine visit cost per sow per year - herd size 50 sows (CHF)**	19.00	19.00	0.00	[a,b]Calculated: the flat-rate fee is 200.- per herd, plus 15.- per animal,<br>[c]set to zero.
**Veterinary routine visit cost per sow per year - herd size 150 sows (CHF)**	16.33	16.33	0.00	[a,b]Calculated: the flat-rate fee is 200.- per herd, plus 15.- per animal,<br>[c]set to zero.
**Iron injection cost (CHF/piglet)**	0.20	0.20	0.20	[a,b,c]Expert opinion.
**Total Iron injection cost (CHF)**	5.64	6.61	8.23	[a,b,c]Calculated: (Iron injection cost * Piglets born alive per year)
**Vaccination cost (CHF/piglet)**	3.00	3.00	0.00	[a,b]Expert opinion,<br>[c]set to zero.
**Total vaccination cost (CHF)**	75.90	91.80	0.00	[a,b,c] Calculated: (Vaccination cost * Piglets weaned per sow per year)
**Piglet castration cost (CHF/piglet)**	1.50	1.50	1.50	[a,b,c]Expert opinion.
**Total cost of piglet castrations (CHF/year)**	18.98	22.95	30.87	[a,b,c]Calculated: (Piglet castration cost * Piglets weaned per sow per year/2)
**Total veterinary costs per per sow per year - herd size 20 sows (CHF)**	125.52	146.36	39.11	[a,b,c]Calculated: (Veterinary routine visitcost + Total Iron injection cost + Total vaccination cost + Total cost of piglet castrations)
**Total veterinary costs per per sow per year - herd size 50 sows (CHF)**	119.52	140.36	39.11	[a,b,c]Calculated: (Veterinary routine visitcost + Total Iron injection cost + Total vaccination cost + Total cost of piglet castrations)
**Total veterinary costs per per sow per year - herd size 150 sows (CHF)**	116.85	137.69	39.11	[a,b,c]Calculated: (Veterinary routine visitcost + Total Iron injection cost + Total vaccination cost + Total cost of piglet castrations)
**Miscellaneous costs**	**Average[a]**	**Top10[b]**	**Ideal[c]**	
Fattening farms				
**Membership costs for producer organisations (e.g. Suisseporcs, Qualiporcs) (CHF/pig)**	1.00	1.00	1.00	[a,b,c]Agridea, 2023
**Cleaning costs fattening farms (CHF/pig)**	6.00	6.00	6.00	[a,b,c]Agridea, 2023
**Transport costs farms (CHF/pig)**	16.00	16.00	16.00	[a,b,c]Agridea, 2023
**Energy costs farms (CHF/pig)**	3.00	3.00	3.00	[a,b,c]Agridea, 2023
**Water costs farms (CHF/pig)**	1.00	1.00	1.00	[a,b,c]Agridea, 2023

**Label inspection costs fattening farms (CHF/pig)**	1.00	1.00	1.00	[a,b,c]Agridea, 2023
Breeding farms				
**Membership costs for producer organisations (e.g. Suisseporcs, Qualiporcs) (CHF/sow)**	11.00	11.00	11.00	[a,b,c]Agridea, 2023
**Cleaning costs breeding farm (CHF/sow)**	13.00	13.00	13.00	[a,b,c]Agridea, 2023
**Transport costs breeding farms (CHF/sow)**	201.00	201.00	201.00	[a,b,c]Agridea, 2023
**Energy costs breeding farms (CHF/sow)**	50.00	50.00	50.00	[a,b,c]Agridea, 2023
**Water costs breeding farms (CHF/sow)**	6.00	6.00	6.00	[a,b,c]Agridea, 2023
**Label inspections costs breeding farms (CHF/sow)**	2.00	2.00	2.00	[a,b,c]Agridea, 2023
**Inseminations per sow per year**	2.7	2.5	2.3	[a,b,c]Calculated: (Litters per sow per year / Farrowing rate)
**Average blisters used per insemination**	1.6	1.6	1.6	[a]Calculated: (Insemination blisters used per sow per year/ Inseminations per sow per year),<br>[b,c]Value from the Average scenario used.
**Insemination blisters used per sow per year**	4.30	4.09	3.8	[a]Agridea, 2023,<br>[b,c]Calculated: (Inseminations per sow per year * Average blisters used per insemination)
**Insemination blister cost (CHF/piece)**	7.80	7.80	7.80	[a,b,c]Agridea, 2023
**Ear tag cost (CHF/piglet)**	0.33	0.33	0.33	[a,b,c]Agridea, 2023
**Total ear tag costs (CHF)**	9.31	10.90	13.58	[a,b,c]Calculated: (Piglets born alive per year * Ear tag cost)

Part 3 – Ideal scenario Monte Carlo simulated parameters.

For the ideal scenario, seven parameters are simulated using a Monte Carlo simulation based on a PERT distribution. These are:

1. Duration of fattening,
2. Feed conversion ratio for fattening pigs
3. Rearing piglet average daily weight gain
4. Feed conversion ratio for rearing piglets
5. Sow replacement rate
6. Litters per sow per year
7. Piglets born alive per litter

All of these parameters except for litters per sow per year are based on a percentage improvement on the value for Top10 farms. Thus, the most likely value is set to 10% 'better' than the value for Top10 farms, and the minimum and maximum values are 5% and 15% better. For parameters where a lower value is considered better (duration of fattening, feed conversion ratio, sow replacement rate), the most likely value is 10% lower than the Top10 farm value, the minimum is 15% lower, and the maximum is 5% lower. For parameters where a higher value is considered better (average daily weight gain, piglets born alive per litter), the most likely value is 10% higher than the Top10 farm value, the minimum is 5% higher, and the maximum is 15% higher.

Using 5%, 10%, and 15% improvements on the Top10 was not possible for litters per sow per year, based on the assumption that the lowest possible achievable weaning to oestrus interval for Swiss production is 7d. Therefore, we set the upper limit (maximum) for the PERT distribution to 2.37, which corresponds to the maximum achievable litters per sow per year when weaning to oestrus interval, lactation and gestation are fixed to 7 days, 31.6 days and 115 days per cycle. This corresponds to 2.5% higher value compared to Top10 farms. Such that the PERT distribution is symmetrical and always exceeds the values achieved by Top10 farms, we set the minimum value to the same level as Top10 farms, and most likely to be 1.25% higher than Top10 farms.

References

1. Gilbert W, Marsh TL, Chaters G, Jemberu WT, Bruce M, Steeneveld W, et al. Quantifying cost of disease in livestock: a new metric for the Global Burden of Animal Diseases. Lancet Planet Health. 2024 May;8(5):e309–17.

2. Soede NM, Langendijk P, Kemp B. Reproductive cycles in pigs. Anim Reprod Sci. 2011 Apr;124(3–4):251–8.

3. Vose D. Risk Analysis – A Quantitative Guide. 2008. 752 pages.
